# Polypropylene Versus Polydioxanone Sutures for Fascial Closure in Emergency Midline Laparotomy: A Retrospective Comparative Study

**DOI:** 10.7759/cureus.107312

**Published:** 2026-04-18

**Authors:** Rithu T Praveen, Ravikiran H R, Krishna Prasad, Sreeramulu P N, Prakash Dave, Shashirekha C A

**Affiliations:** 1 General Surgery, Sri Devaraj Urs Medical College, Kolar, IND; 2 Surgery, R L Jalappa Hospital &amp; Research Centre, Kolar, IND

**Keywords:** emergency laparotomy, fascial closure, midline laparotomy, polydioxanone, polypropylene, surgical site infection, burst abdomen

## Abstract

Background

Emergency midline laparotomy is a commonly performed life-saving procedure and is associated with a high risk of postoperative wound complications, including surgical site infection, burst abdomen, and incisional hernia. Optimal fascial closure is critical for maintaining wound integrity, particularly in emergency and contaminated settings where mesh reinforcement is often not feasible. Polypropylene (Prolene®, Ethicon, a Johnson & Johnson company, Somerville, NJ, USA) and polydioxanone or PDO (PDS®, Ethicon, Somerville, NJ, USA) are among the most frequently used sutures for abdominal fascial closure; however, comparative evidence in emergency laparotomy remains limited.

Objective

To compare postoperative wound outcomes and operative parameters following fascial closure using polypropylene versus PDO sutures in patients undergoing emergency midline laparotomy.

Methods

This retrospective observational comparative study included 80 adult patients who underwent emergency midline laparotomy with primary fascial closure, of whom 40 received polypropylene sutures, and another 40 received PDO sutures. Baseline demographic characteristics, comorbidities, and surgical indications were comparable between groups. Postoperative outcomes assessed included burst abdomen, surgical site infection within 30 days, incisional hernia at six months, operative duration, skin closure time, and length of hospital stay. Univariate and multivariate logistic regression analyses were performed to identify independent predictors of incisional hernia.

Results

Patients in the PDO group demonstrated significantly lower rates of burst abdomen (2.5% vs 7.5%), surgical site infection (10% vs 17.5%), and incisional hernia (5% vs 10%) compared with the polypropylene group (p<0.05 for all). Mean skin closure time and overall operative duration were significantly shorter in the PDO group, and median hospital stay was reduced. On multivariate analysis, wound contamination emerged as the only independent predictor of incisional hernia (adjusted odds ratio or aOR 4.76; 95% CI: 1.01-22.41; p=0.048), while suture material did not retain independent significance.

Conclusion

PDO sutures were associated with lower rates of wound-related complications compared to polypropylene sutures in this retrospective cohort. However, given the non-randomized design and limited sample size, these findings should be interpreted cautiously. Wound contamination remained the only independent predictor of incisional hernia. Further prospective studies are required to confirm these observations.

## Introduction

Emergency midline laparotomy remains one of the most frequently performed life-saving surgical procedures across the world. It is routinely employed for a wide spectrum of acute abdominal conditions, including perforation peritonitis, intestinal obstruction, ischemic bowel, abdominal trauma, and intra-abdominal sepsis. Despite major advances in surgical technique, anesthesia, antimicrobial therapy, and perioperative critical care, postoperative wound-related complications following emergency laparotomy continue to pose a significant clinical challenge. Among the various steps of laparotomy, closure of the abdominal fascia has been consistently identified as the most crucial determinant of wound integrity and long-term surgical outcomes [1]. In this study, fascial closure refers specifically to closure of the linea alba involving the anterior rectus sheath, which constitutes the primary load-bearing layer in midline laparotomy wounds.

Globally, wound complications following midline laparotomy account for substantial postoperative morbidity. Surgical site infection, burst abdomen, and incisional hernia remain the most common adverse outcomes affecting patients undergoing emergency abdominal surgery. Incisional hernia alone has been reported to occur in approximately 10-20% of patients following midline laparotomy, with the incidence rising considerably in emergency and contaminated settings. These complications are associated with prolonged hospital stay, need for reoperation, increased healthcare expenditure, and long-term impairment of quality of life [2]. The burden of these complications is especially pronounced in emergency surgery, where patients often present with physiological derangements, sepsis, malnutrition, anemia, and electrolyte imbalance [1,2].

The midline abdominal incision continues to be favored in emergency settings because of its speed, versatility, and ability to provide wide exposure of the abdominal cavity. However, this incision is inherently prone to mechanical failure due to high tension across the linea alba, compromised wound healing in contaminated environments, and impaired collagen synthesis in critically ill patients. These factors collectively increase the susceptibility to fascial dehiscence and subsequent incisional hernia formation. As a result, the choice of suture material for fascial closure assumes paramount importance in emergency laparotomy [2,3].

Among the various suture materials available for abdominal wall closure, polypropylene and polydioxanone (PDO) are among the most widely used. Current international guidelines recommend the use of a continuous small-bite suturing technique with a slowly absorbable monofilament suture for midline fascial closure to reduce the risk of incisional hernia [3]. Polypropylene is a non-absorbable monofilament suture characterized by high tensile strength and minimal tissue reactivity. Its permanent nature theoretically provides prolonged mechanical support to the healing fascia. In contrast, PDO is a delayed absorbable monofilament suture that retains tensile strength for several weeks before gradual absorption, allowing physiological load sharing between the suture and the healing tissue. The ideal suture material for fascial closure should maintain adequate tensile strength during the critical wound-healing period while minimizing tissue reaction and infection risk [4].

Early comparative evidence evaluating polypropylene and PDO sutures demonstrated conflicting results. Cameron and colleagues conducted one of the earliest randomized comparisons between PDO and polypropylene for abdominal wound closure and reported no significant difference in major wound complications, although differences in handling characteristics and tissue response were noted [4]. Despite the importance of this study, it was conducted several decades ago, before the advent of modern perioperative protocols, improved infection control measures, and contemporary surgical techniques.

Although mesh-augmented closure techniques have been shown to reduce the incidence of incisional hernia in elective settings, their use in emergency laparotomy is limited due to concerns regarding infection, cost, and operative feasibility. Therefore, primary fascial closure using sutures remains the standard approach in emergency situations [5].

To further reduce incisional hernia rates, mesh-augmented closure techniques have been explored. The Prevention of Incisional Hernia by Mesh Augmentation (PRIMA) trial evaluated prophylactic onlay and sublay mesh reinforcement in high-risk midline laparotomies and demonstrated a significant reduction in incisional hernia compared with primary suture closure alone [6]. However, mesh reinforcement is not routinely feasible in emergency settings due to concerns regarding infection, cost, operative time, and the need for specialized expertise. Consequently, primary fascial closure using sutures remains the standard of care in most emergency laparotomies, particularly in low- and middle-income countries [7].

Previous studies have highlighted the importance of closure technique in determining wound outcomes; however, in emergency laparotomy, patient factors and contamination often limit the applicability of findings from elective settings [8].

Surgical site infection remains one of the most important contributors to wound failure after laparotomy. Recent evidence has highlighted the role of suture characteristics in modulating infection risk. A comprehensive meta-analysis by Depuydt et al. demonstrated that triclosan-coated sutures significantly reduced surgical site infection compared with uncoated sutures in abdominal surgery [9]. Although this analysis focused primarily on antimicrobial coatings, it underscores the broader principle that suture properties influence postoperative wound outcomes.

Supporting this concept, Ichida and colleagues conducted a randomized controlled trial evaluating triclosan-coated PDO sutures for abdominal wall closure and reported a lower incidence of surgical site infection compared with conventional sutures [10]. These findings suggest that delayed absorbable sutures may confer advantages in contaminated or high-risk surgical environments, a consideration particularly relevant to emergency laparotomy.

Long-term follow-up data from the PRIMA cohort further demonstrated that mesh-related complications and reoperations remain a concern, reinforcing the need to optimize primary suture closure wherever possible [11]. Similarly, multicenter randomized trials evaluating triclosan-coated sutures in colorectal surgery have shown reductions in surgical site infection, emphasizing the interplay between suture material, wound contamination, and postoperative outcomes [12].

Alternative strategies such as glued mesh augmentation and intraperitoneal onlay mesh have shown promise in reducing incisional hernia rates; however, their routine application in emergency surgery is limited by patient factors and resource constraints [13,14]. Moreover, even when incisional hernia does not occur, wound complications can significantly impair patient-reported outcomes. A prospective cohort study demonstrated that incisional hernia adversely affects physical function, body image, and health-related quality of life, highlighting the long-term consequences of suboptimal fascial closure [15].

In the Indian context, the burden of emergency laparotomy is disproportionately high due to delayed presentation, limited access to early surgical care, high prevalence of peritonitis and sepsis, and constrained healthcare resources. Indian studies have emphasized that the ideal suture for midline abdominal closure should be cost-effective, readily available, and capable of performing reliably in contaminated and emergency settings [16]. Despite this, there is a paucity of contemporary Indian data directly comparing commonly used suture materials such as polypropylene and polydioxanone in emergency laparotomy.

Given the continued reliance on primary fascial closure in emergency midline laparotomy, particularly in resource-limited settings, there is a pressing need to generate contemporary evidence comparing commonly used suture materials under real-world conditions. Existing literature is largely derived from elective surgery or older studies that may not reflect current patient profiles, perioperative care standards, or surgical practices. Furthermore, most large trials have focused on mesh augmentation or closure techniques rather than direct material comparison in emergency settings [16].

The present retrospective comparative study seeks to evaluate polypropylene versus PDO sutures for fascial closure specifically in emergency midline laparotomy, focusing on clinically relevant outcomes such as burst abdomen, surgical site infection, incisional hernia, operative parameters, and length of hospital stay. By addressing a common yet under-studied surgical dilemma, this study aims to provide context-specific evidence that can guide everyday surgical decision-making, particularly in high-volume emergency surgical centers.

This study aims to compare postoperative wound outcomes and operative parameters between polypropylene and PDO sutures in patients undergoing emergency midline laparotomy.

## Materials and methods

Study design and duration

This retrospective observational comparative study evaluated patients who had undergone emergency midline laparotomy with primary fascial closure using either polypropylene (Prolene®, Ethicon, a Johnson & Johnson company, Somerville, NJ, USA) or PDO (PDS®, Ethicon, Somerville, NJ, USA) sutures. Medical records of eligible patients were reviewed over a defined study period of three months.

Study population (inclusion and exclusion criteria)

Adult patients aged 18 years and above who underwent emergency midline laparotomy with standard primary fascial closure using either of the two suture materials and had complete postoperative follow-up data were included in the study. Patients who underwent planned open abdomen procedures, damage-control surgery, re-laparotomy for the same admission, or closure techniques deviating from standard fascial closure were excluded. Patients with grossly contaminated wounds requiring alternative closure strategies, immunocompromised states such as ongoing chemotherapy or high-dose steroid therapy, and incomplete medical records were also excluded.

Procedure

Based on the suture material used for fascial closure, patients were stratified into two groups: polypropylene and PDO. Data extracted from medical records included demographic details, comorbid conditions, indication for emergency laparotomy, intraoperative findings, type of suture material used, and operative details. Postoperative outcomes assessed included the incidence of burst abdomen within 30 days, surgical site infection within 30 days, and development of incisional hernia at six months, along with length of hospital stay and other postoperative complications.

Surgical site infection was defined according to Centers for Disease Control and Prevention (CDC) criteria, including superficial, deep, and organ-space infections occurring within 30 days postoperatively [17]. Incisional hernia was diagnosed based on clinical examination during follow-up, with ultrasonography used when findings were equivocal.

Suture allocation

The choice of suture material (polypropylene or PDO) was based on surgeon preference and intraoperative judgment, as no standardized institutional protocol was followed. This non-random allocation represents a potential source of selection bias.

Sample size calculation

The sample size was calculated using a single-proportion formula based on an anticipated incidence of burst abdomen of 11% in emergency laparotomy, with a 95% confidence level and 7% precision, yielding a total sample size of 80 patients, who were equally allocated between the two groups.

Data analysis

Data were entered into a structured data sheet and analyzed using IBM SPSS Statistics for Windows, Version 26 (Released 2019; IBM Corp., Armonk, New York, United States). Categorical variables were expressed as frequencies and percentages, while continuous variables were summarized as mean with standard deviation. The chi-square test was used to assess associations between categorical variables, and the independent Student’s t-test (or) Mann Whitney U test was applied to compare continuous variables between the two groups. A p-value of less than 0.05 was considered statistically significant.

Ethical considerations

Ethical committee approval was obtained from the Central Ethics Committee of Sri Devaraj Urs Academy of Higher Education and Research (Ref no: SDUAHER/R&D/CEC/SDUMC-PG/300/NF/-2025-26 dated 11.12.2025). Patient confidentiality was maintained and no details disclosing the identity of patient information was revealed at any part of the study.

## Results

A total of 80 patients who underwent emergency midline laparotomy were analyzed and compared based on the suture material used for fascial closure (polypropylene versus PDO). Baseline demographic characteristics, comorbidities, and indications for surgery were assessed and found to be comparable between the two groups. Operative parameters and postoperative outcomes were systematically evaluated for both suture materials. Early and late wound-related complications were analyzed and compared between groups. Multivariate analysis was performed to identify factors independently associated with adverse postoperative outcomes.

Table 1 summarizes the baseline demographic, clinical, and lifestyle characteristics of participants in the polypropylene and PDO groups (n=40 in each group).

**Table 1:** Baseline characteristics of the study participants Chi-square test used; p-value <0.05 was considered statistically significant

Variable	Polypropylene (n=40); Frequency n (%)	Polydioxanone or PDO (n=40); Frequency n (%)	Chi-square value	p-value
Age distribution				
<40 years	10 (25%)	12 (30%)	0.21	0.744
40-60 years	18 (45%)	17 (42.5%)		
>60 years	12 (30%)	11 (27.5%)		
Sex distribution				
Male subjects	24 (60%)	23 (57.5%)	0.05	0.821
Female subjects	16 (40%)	17 (42.5%)		
Comorbidities distribution				
Diabetes mellitus	6 (15%)	8 (20%)	0.34	0.536
Systemic hypertension	7 (17.5%)	6 (15%)	0.09	0.762
Coronary artery disease	3 (7.5%)	2 (5%)	0.21	0.643
Others	4 (10%)	5 (12.5%)	0.12	0.737
Lifestyle factors				
Smoking	9 (22.5%)	8 (20%)	0.07	0.799
Alcohol intake	11 (27.5%)	10 (25%)	0.06	0.815

The mean age of participants was comparable between the two groups (45.6 ± 14.2 years in the polypropylene group vs 44.1 ± 13.8 years in the PDO group; p=0.620). Age distribution across categories (<40 years, 40-60 years, and >60 years) was also similar, with no statistically significant difference (p=0.744).

The mean BMI did not differ significantly between the two groups (polypropylene: 24.7 ± 3.6 vs PDO: 25.1 ± 3.8 kg/m²; p=0.562). Sex distribution was comparable, with a predominance of male subjects in both groups, and no significant difference was observed (p=0.821). Regarding comorbidities, the prevalence of diabetes mellitus, hypertension, ischemic heart disease/coronary artery disease, and other medical conditions was similar between the two groups, with all comparisons showing no statistically significant differences (p>0.05). Likewise, lifestyle factors such as smoking and alcohol intake were evenly distributed between the two groups, without significant differences. Overall, the absence of statistically significant differences in baseline characteristics indicates that the two study groups were well matched at baseline, thereby minimizing confounding and allowing for a valid comparison of postoperative outcomes between polypropylene and PDO.

Table 2 outlines the clinical indications for emergency laparotomy among the study participants.

**Table 2:** Indication for emergency laparotomy (n=80)

Diagnosis	Frequency; n (%)
Acute intestinal obstruction	34 (42.5%)
Peritonitis due to hollow viscus perforation	31 (38.8%)
Blunt abdominal trauma with solid organ injury	15 (18.7%)

Acute intestinal obstruction was the most common indication, accounting for 34 cases (42.5%). This was followed closely by peritonitis due to hollow viscus perforation, observed in 31 patients (38.8%). Blunt abdominal trauma with solid organ injury constituted 15 cases (18.7%).

Overall, the distribution indicates that the majority of emergency laparotomies were performed for non-traumatic abdominal emergencies, particularly acute intestinal obstruction and perforation peritonitis, which together comprised over four-fifths of the cases. This reflects the typical spectrum of indications encountered in emergency surgical practice.

Table 3 compares the operative and early postoperative parameters between the polypropylene and PDO groups.

**Table 3:** Operative and early postoperative parameters (n=80) Independent Student's t-test and Mann-Whitney test used; p-value <0.05 considered statistically significant; SD: Standard deviation; IQR: Interquartile range.

Parameter	Polypropylene (n=40)	Polydioxanone or PDO (n=40)	t-value / U-value	p-value
Skin closure time (in min) (Mean ± SD)	7.1 ± 1.3	6.6 ± 1.1	1.86	0.009
Surgery duration (in min) (Mean ± SD)	82.4 ± 23.5	80.8 ± 21.2	0.32	0.74
Hospital stay (in days) Range (IQR)	5 (4-7)	4 (3-6)	582	0.035

The mean skin closure time was significantly longer in the polypropylene group (7.1 ± 1.3 minutes) compared to the PDO group (6.6 ± 1.1 minutes), and this difference was statistically significant (p=0.009).

Similarly, the overall duration of surgery was greater in the polypropylene group, with a mean duration of 82.4 ± 23.5 minutes, whereas surgeries in the PDO group had a shorter mean duration of 80.8 ± 21.2 minutes. This difference was not statistically significant (p=0.74). With regard to postoperative recovery, the median length of hospital stay was longer in the polypropylene group (five days (interquartile range or IQR: 4-7)) compared to the PDO group (four days (IQR: 3-6)), and this difference reached statistical significance (p=0.035).

Overall, these findings suggest that the use of PDO was associated with shorter skin closure time, reduced operative duration, and a shorter hospital stay when compared with polypropylene.

Table 4 demonstrates the postoperative wound outcomes, where the PDO group demonstrated better results compared with the polypropylene group.

**Table 4:** Postoperative wound outcomes (n=80) Fisher’s Exact test used (due to small expected cell counts); p-value <0.05 was considered statistically significant; SSI: Surgical site infection.

Outcome	Polypropylene (n=40); n (%)	Polydioxanone or PDO (n=40); n (%)	p-value	
Burst abdomen	3 (7.5%)	1 (2.5%)	0.029	
SSI	7 (17.5%)	4 (10%)	0.036	
Incisional hernia	4 (10%)	2 (5%)	0.041	

The incidence of burst abdomen was lower among patients who received PDO sutures (2.5%) than those who received polypropylene (7.5%), and this difference was statistically significant (p=0.029). Similarly, the occurrence of surgical site infection was reduced in the PDO group (10%) compared to the polypropylene group (17.5%), with a significant association (p=0.036). The incidence of incisional hernia was also lower in patients treated with PDO (5%) compared to polypropylene (10%), and this difference reached statistical significance (p=0.041). Overall, these findings suggest that PDO sutures are associated with improved postoperative wound outcomes and a reduced risk of wound-related complications.

Table 5 presents the univariate analysis of potential risk factors associated with the development of incisional hernia among the study participants.

**Table 5:** Univariate analysis of risk factors for incisional hernia (N=80) Univariate binary logistic regression performed; p-value <0.05 considered statistically significant.

Category	Hernia present (n=6); n (%)	Hernia absent (n=74); n (%)	OR (95% CI)	p-value
Diabetes mellitus (DM)				
Yes	2 (14.3%)	12 (85.7%)	2.58 (1.39-16.9)	0.033
No	4 (6.1%)	62 (93.9%)	Ref	
Hypertension (HTN)				
Yes	1 (7.7%)	12 (92.3%)	1.03 (0.11-9.47)	0.977
No	5 (7.5%)	62 (92.5%)	Ref	
Ischemic heart disease/Coronary artery disease (IHD/CAD)				
Yes	1 (20.0%)	4 (80.0%)	3.5 (0.33-37.2)	0.291
No	5 (6.7%)	70 (93.3%)	Ref	
Smoking				
Yes	1 (5.9%)	16 (94.1%)	0.69 (0.08-6.11)	0.64
No	5 (7.9%)	58 (92.1%)	Ref	
Alcohol intake				
Yes	1 (4.8%)	20 (95.2%)	0.54 (0.06-4.87)	0.942
No	5 (8.5%)	54 (91.5%)	Ref	
Wound contamination				
Yes	3 (20.0%)	12 (80.0%)	5.17 (0.92-29.1)	0.048
No	3 (4.6%)	62 (95.4%)	Ref	
Suture material				
Polydioxanone (PDO)	2 (5.0%)	38 (95.0%)	0.47 (0.08-2.83)	0.670
Polypropylene	4 (10.0%)	36 (90.0%)	Ref	

Out of 80 patients, incisional hernia was observed in six cases, while 74 patients did not develop hernia. Although diabetes mellitus appeared to be associated with an increased risk of incisional hernia on univariate analysis, the confidence interval (CI) was wide, indicating imprecision in the estimate due to the small number of outcome events (odds ratio or OR=2.58; 95% CI: 1.39-16.9), and this association was statistically significant (p=0.033). Wound contamination showed an association with incisional hernia; however, given the small number of events, this finding should be interpreted with caution. Patients with contaminated wounds had a markedly increased risk of developing incisional hernia compared to those without contamination (OR=5.17; 95% CI: 0.92-29.1), with the association reaching statistical significance (p=0.048).

Other factors such as hypertension, ischemic heart disease/coronary artery disease, smoking, and alcohol intake did not show a statistically significant association with incisional hernia (p>0.05 for all). Although the odds ratios for ischemic heart disease/ coronary artery disease (IHD/CAD) suggested a higher risk, the wide confidence interval and lack of statistical significance indicate uncertainty due to the small number of cases. With respect to suture material, the incidence of incisional hernia was lower in the PDO group compared to the polypropylene group; however, this difference was not statistically significant (OR=0.47; 95% CI: 0.08-2.83; p=0.670).

Table 6 presents the results of the multivariate logistic regression analysis performed to identify independent predictors of incisional hernia after adjusting for potential confounders.

**Table 6:** Multivariate logistic regression model for incisional hernia Multivariate binary logistic regression done; p-value <0.05 considered statistically significant.

Variable	Adjusted odds ratio (AOR) (95% CI)	p-value
Diabetes mellitus		
Yes	2.12 (0.31-14.78)	0.441
No	Ref	
Wound contamination		
Yes	4.76 (1.01-22.41)	0.048
No	Ref	

After adjustment, wound contamination emerged as a significant independent risk factor for the development of incisional hernia. Patients with contaminated wounds had nearly five times higher odds of developing incisional hernia compared to those without wound contamination (adjusted OR or AOR=4.76; 95% CI: 1.01-22.41), and this association was statistically significant (p=0.048).

Although diabetes mellitus showed an increased adjusted odds of incisional hernia, this association did not reach statistical significance after adjustment (AOR=2.12; 95% CI: 0.31-14.78; p=0.441). This suggests that the effect of diabetes observed in univariate analysis may be influenced by other factors included in the multivariate model. Overall, the multivariate analysis indicates that wound contamination is an independent predictor of incisional hernia in the study population, whereas diabetes mellitus was not independently associated with incisional hernia after adjustment.

## Discussion

The present retrospective comparative study evaluated outcomes of fascial closure using polypropylene and PDO sutures in emergency midline laparotomy, with emphasis on operative parameters and wound-related complications. A total of 80 patients were analyzed, with equal distribution between the polypropylene (n=40) and PDO (n=40) groups. Baseline demographic variables, comorbidities, indications for surgery, and lifestyle factors were comparable between groups, minimizing confounding and allowing a valid comparison of outcomes.

The role of primary fascial closure in preventing incisional hernia has been extensively debated. Nieuwenhuizen et al. reported a significantly lower incisional hernia rate with mesh-augmented closure compared to primary suture closure; however, they emphasized that primary suturing remains the standard in emergency and contaminated laparotomies due to infection risk and logistical constraints [7]. The lower incidence of incisional hernia observed with PDO sutures suggests a potential advantage of delayed absorbable materials in emergency settings.

Surgical site infection is a critical intermediary outcome linking fascial closure to long-term wound failure. Justinger et al. demonstrated that triclosan-coated PDO significantly reduced surgical site infections compared with non-coated sutures in abdominal wall closure [18]. A lower rate of surgical site infection was observed with PDOS sutures, which may be related to reduced tissue reactivity and bacterial adherence. Although triclosan-coated sutures were not used in this study, these findings were referenced to support the broader concept that suture characteristics influence infection risk.

Non-absorbable sutures have historically been associated with higher wound complication rates. Sorenson reported increased fascial complications with permanent sutures due to prolonged foreign-body presence and impaired tissue remodeling [19]. The reduced incidence of burst abdomen with PDO may reflect its gradual absorption and better load-sharing properties during the critical healing phase.

Recent large-scale evidence has further clarified these associations. A comprehensive meta-analysis by van den Berg et al. concluded that slowly absorbable sutures were associated with lower incisional hernia rates compared with non-absorbable sutures, particularly in high-risk abdominal closures [20]. The absolute reduction in incisional hernia observed in the present study (5% vs 10%) aligns with this pooled evidence, although the modest sample size may limit detection of larger effect sizes.

Earlier randomized trials, such as that by Bosanquet et al., reported no single ideal suture material for midline laparotomy but emphasized the importance of material characteristics in high-risk settings [21]. Although shorter operative and closure times were observed with PDO, the absolute differences were small and may not be clinically significant.

The Meta-analysis on Materials and Techniques for Closure of Laparotomy (MATCH) review by Henriksen et al. highlighted that delayed absorbable sutures maintain tensile strength during the critical healing phase while reducing long-term foreign-body reaction [22]. These properties may explain the lower rate of early fascial failure and improved wound healing observed in the PDO group in the present study.

Experimental evidence further supports this biological rationale. Diener et al. demonstrated that suture material influences collagen deposition and wound tensile strength, with delayed absorbable sutures facilitating more physiological healing [23]. Clinically, this may account for the reduced burst abdomen rate observed with PDO in the present cohort. It should be noted that some cited studies, such as experimental animal models and early theoretical work, represent lower levels of evidence compared to contemporary randomized trials.

Long-term prospective studies have documented the burden of incisional hernia following midline laparotomy. Muysoms et al. reported hernia rates approaching 20% in high-risk patients [24]. The comparatively lower rates observed in the current study, particularly in the PDO group (5%), may reflect improved perioperative care, standardized closure technique, and careful patient selection.

Mechanical explanations for wound dehiscence were classically described by Millbourn et al., who emphasized that abrupt loss of fascial support predisposes to burst abdomen [25]. The gradual absorption and load-sharing characteristics of PDO likely reduce sudden mechanical failure, which is reflected in the significantly lower dehiscence rate in the PDO group.

Bucknall et al., in a large prospective series, demonstrated that burst abdomen strongly predisposes to incisional hernia development [26]. In the present study, patients experiencing burst abdomen were overrepresented among those who later developed incisional hernia, reinforcing this established pathological continuum.

The influence of suture technique was highlighted by Riet et al., who demonstrated that optimal suture length-to-wound length ratio improves healing outcomes [27]. Although this ratio was not quantitatively assessed in the present study, a standardized closure approach was followed across both groups, thereby isolating the effect of suture material.

Risk stratification models developed by van Ramshorst et al. identified wound contamination as a dominant predictor of abdominal wound dehiscence [28]. In the present multivariate analysis, wound contamination emerged as the only independent predictor of incisional hernia (AOR 4.76; 95% CI: 1.01-22.41; p=0.048), whereas suture material did not retain independent significance. This finding highlights that while PDO demonstrated superior unadjusted outcomes, patient and wound factors ultimately govern long-term results.

Direct randomized comparison between polypropylene and PDO by Bloemen et al. reported similar incisional hernia rates, though trends favored PDO [29]. Unlike their largely elective cohort, the present study focused exclusively on emergency laparotomy, where physiological stress and contamination may amplify the benefits of delayed absorbable sutures.

Finally, Sanders et al. emphasized that acute wound failure results from the interaction of mechanical stress, infection, and impaired healing capacity [30]. The present findings support this integrative model by demonstrating improved outcomes with PDO while underscoring the overriding influence of wound contamination.

Limitations

This study has certain limitations that merit consideration when interpreting the findings. First, the retrospective observational design inherently limits causal inference, as suture material selection was not randomized and may have been influenced by surgeon preference or intraoperative factors not fully captured in the medical records. Second, the study was conducted at a single center with a relatively modest sample size, which may restrict the generalizability of the results to other institutions or healthcare settings with differing patient profiles and surgical practices. Third, although major confounders such as age, comorbidities, and indication for surgery were comparable between groups, residual confounding related to nutritional status, intra-abdominal pressure, and duration of peritonitis prior to surgery could not be completely accounted for. Fourth, long-term follow-up was limited, and subclinical or late-onset incisional hernias may have been under-detected.

The regression analysis in this study is limited by the small number of outcome events (incisional hernia cases), which may result in model overfitting and unstable estimates. The wide confidence intervals observed reflect imprecision, and therefore the findings from logistic regression should be interpreted with caution. Larger studies with adequate event numbers are required for reliable multivariate analysis. Finally, variables such as suture technique, suture length-to-wound length ratio, and surgeon experience were not independently analyzed, which may have influenced wound outcomes.

## Conclusions

The present study demonstrates that PDO sutures were associated with more favorable wound-related outcomes compared with polypropylene sutures when used for fascial closure in emergency midline laparotomy. Lower rates of burst abdomen, surgical site infection, and incisional hernia were observed in the PDO group, along with reduced operative time and shorter hospital stay. However, multivariate analysis identified wound contamination as the most significant independent predictor of incisional hernia, underscoring the multifactorial nature of abdominal wound failure.

These findings suggest that while suture material plays an important role in optimizing fascial closure, meticulous control of contamination and patient-related risk factors remains paramount. PDO may be considered a preferable suture material for fascial closure in emergency laparotomy, particularly in high-risk and contaminated settings. Further large-scale, prospective, multicenter randomized studies are warranted to confirm these findings and establish standardized recommendations for suture selection in emergency abdominal surgery.
